# Supplementary material for: Association Between Incident Chronic Kidney Disease and Body Size Phenotypes in Apparently Healthy Adults: An Observational Study Using the Korean National Health and Nutrition Examination Survey (2019–2021)
Source: Biomedicines. 2025 Aug 3;13(8):1886. doi: 10.3390/biomedicines13081886 (PMC12383503; doi:10.3390/biomedicines13081886)
Supplement: Supplementary file 1 [file biomedicines-13-01886-s001.zip › biomedicines-3763405-supplementary.pdf]

Supplement Table S1. Variance inflation factors (VIFs) for covariates included in the logistic regression model.

Variable	VIF	Tolerance
Carbohydrates	1.549	0.646
Protein	2.984	0.335
Fat	2.283	0.438
Total energy	1.647	0.607
Income quartile	1.302	0.768
Urban residence	1.069	0.935
Physical activity	1.047	0.955
Alcohol use	1.174	0.852
Education level	1.703	0.587
Smoking status	1.512	0.661
Sex	1.684	0.594
Age	1.795	0.557

All VIF values were below 3.0, indicating no significant multicollinearity.

Supplement Table S2. Comorbid conditions of the participants excluded from this study

Comorbid Condition*	No CKD (n=8757)	CKD (n=1,446)	P-value
Hypertension (n=8,583)	3,425 (47.6)	1,024 (73.6)	<0.001
Dyslipidemia (n=8,581)	2,998 (41.7)	621 (44.7)	0.021
Diabetes Mellitus (n=10,203)	1,274 (14.5)	559 (38.7)	<0.001
Stroke (n=8,014)	255 (3.8)	105 (8.5)	<0.001
Myocardial Infarction (n=8,007)	144 (2.1)	46 (3.7)	<0.001
Angina Pectoris (n=8,007)	283 (4.2)	69 (5.6)	0.034
Tuberculosis (n=8,006)	481 (7.1)	71 (5.7)	0.087
Asthma (n=9,619)	483 (5.8)	73 (5.6)	0.898
Thyroid Disease (n=8,006)	615 (9.1)	65 (5.2)	<0.001
Malignancy (n=10,203)	706 (8.1)	143 (9.9)	0.022

*Due to missing data in individual variables, the total number of participants (n) varies across comorbid conditions.
